# Supplementary material for: Notch signalling influences cell fate decisions and HOX gene induction in axial progenitors
Source: Development. 2024 Feb 12;151(3):dev202098. doi: 10.1242/dev.202098 (PMC10911136; doi:10.1242/dev.202098)
Supplement: Supplementary information [file develop-151-202098-s1.pdf]

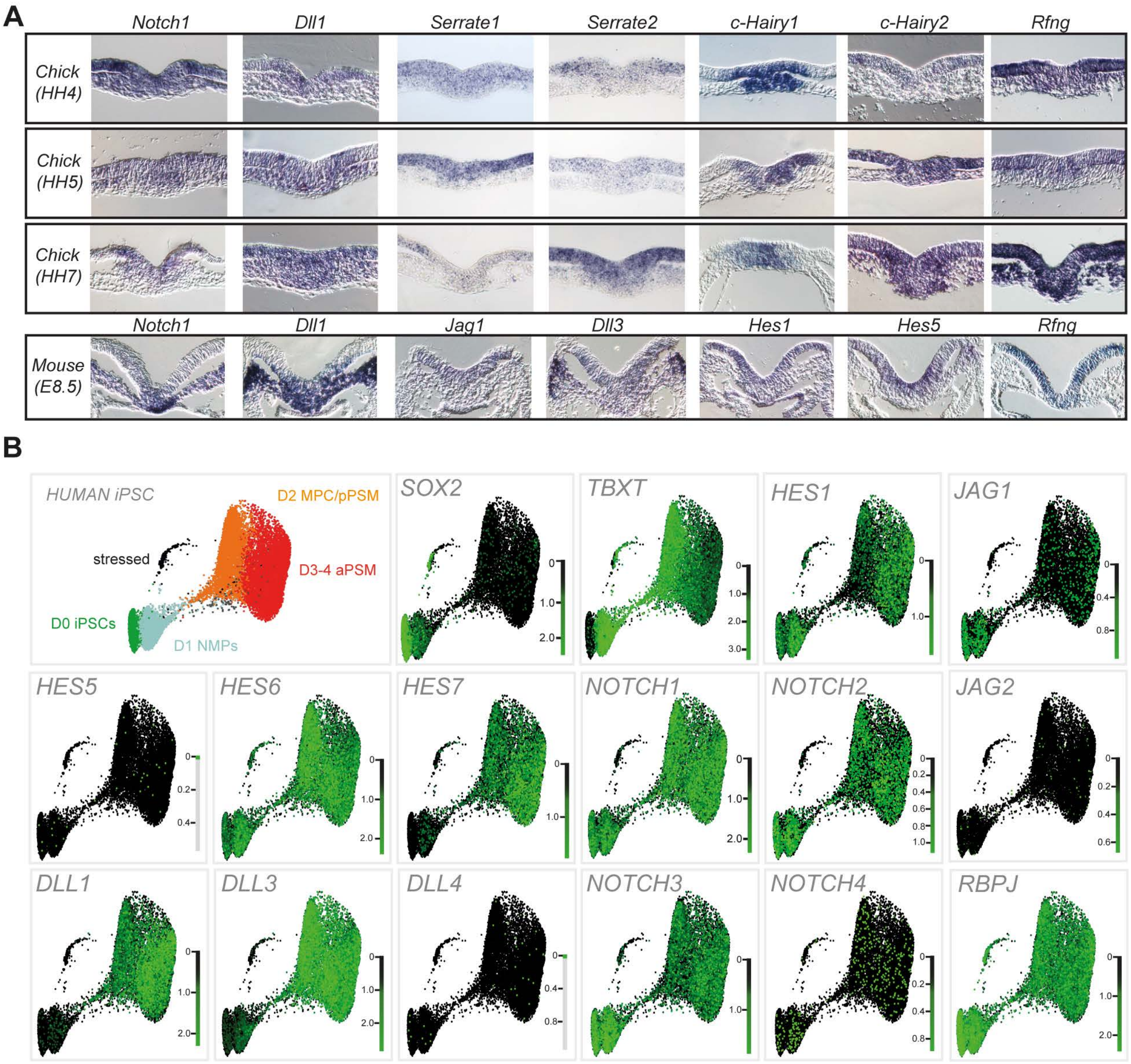

Fig. S1. Expression of Notch signalling components in axial progenitors. (A) *In situ* hybridisation analysis of expression of Notch pathway components in transverse sections of the chick and mouse caudal progenitor area corresponding to the indicated embryonic stages. HH, Hamburger Hamilton, E, embryonic day. (b) ForceAtlas2 layouts of single-cell k-nearest neighbour (kNN) graphs overlaid with log-normalized transcript counts for key components of the Notch pathway in human induced pluripotent stem cells (iPSCs) differentiating toward NMPs and presomitic mesoderm (PSM). Published data obtained from (Diaz-Cuadros et al., 2020). D, differentiation day; aPSM, anterior presomitic mesoderm; pPSM, posterior presomitic mesoderm; MPC, mesodermal precursor cell.

Development • Supplementary information

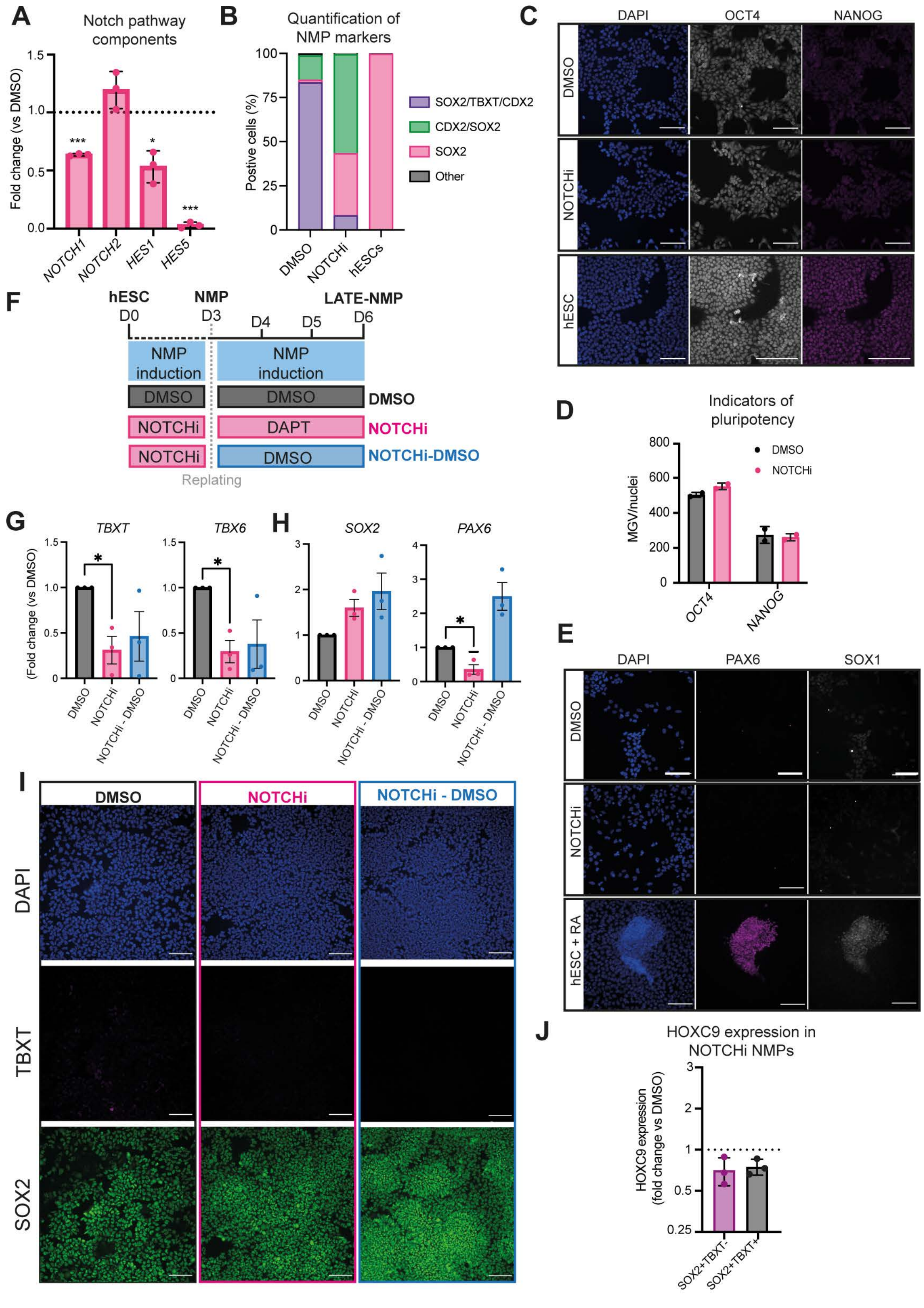

Fig. S2. Effect of Notch signalling inhibition on hESC-derived NMPs. (A) qPCR expression analysis of indicated Notch signalling pathway components/ targets in NOTCHi hESC-derived NMPs compared to DMSO controls. Error bars represent mean±s.e.m n=3. *P≤0.05, **P≤0.01 ***P=≤0.001 (one sample t and Wilcoxon test). (B) Image analysis depicting the percentage of nuclei positive for TBXT, HOXC9 and SOX2 protein expression. Graph shows mean values (n=3

Development • Supplementary information

independent experiments) (C) Pluripotency associated marker expression in DMSO and NOTCHi hESC-derived NMPscompared to undifferentiated hESC controls. (D) Quantification of MGV/nuclei for markers shown in (C). Error bars represent mean±s.d. (n=2 independent experiments). (E) Neuroectoderm associated marker expression in DMSO and NOTCHi hESC-derived NMPs compared to a positive control (hESC+RA). RA, retinoic acid (F) Scheme of depicting Notch manipulation conditions during prolonged NMP culture (G,H) qPCR analysis of *TBXT, TBX6, SOX2* and *PAX6* expression in D6 “late NMP” cultures induced using the conditions shown in (F) Error bars represent mean±s.e.m (n=3 independent experiments). *P≤0.05 (I) Immunofluorescence analysis of the expression of TBXT and SOX2 in “late NMP” cultures generated using the indicated conditions. (J) Immunofluorescence analysis of MGV of HOXC9 and SOX2 protein expression relative to TBXT positivity (TBXT+ or TBXT-) in DAPT-treated NMPs. Error bars represent mean±s.e.m (n=3 independent experiments, paired two-tailed t-test).

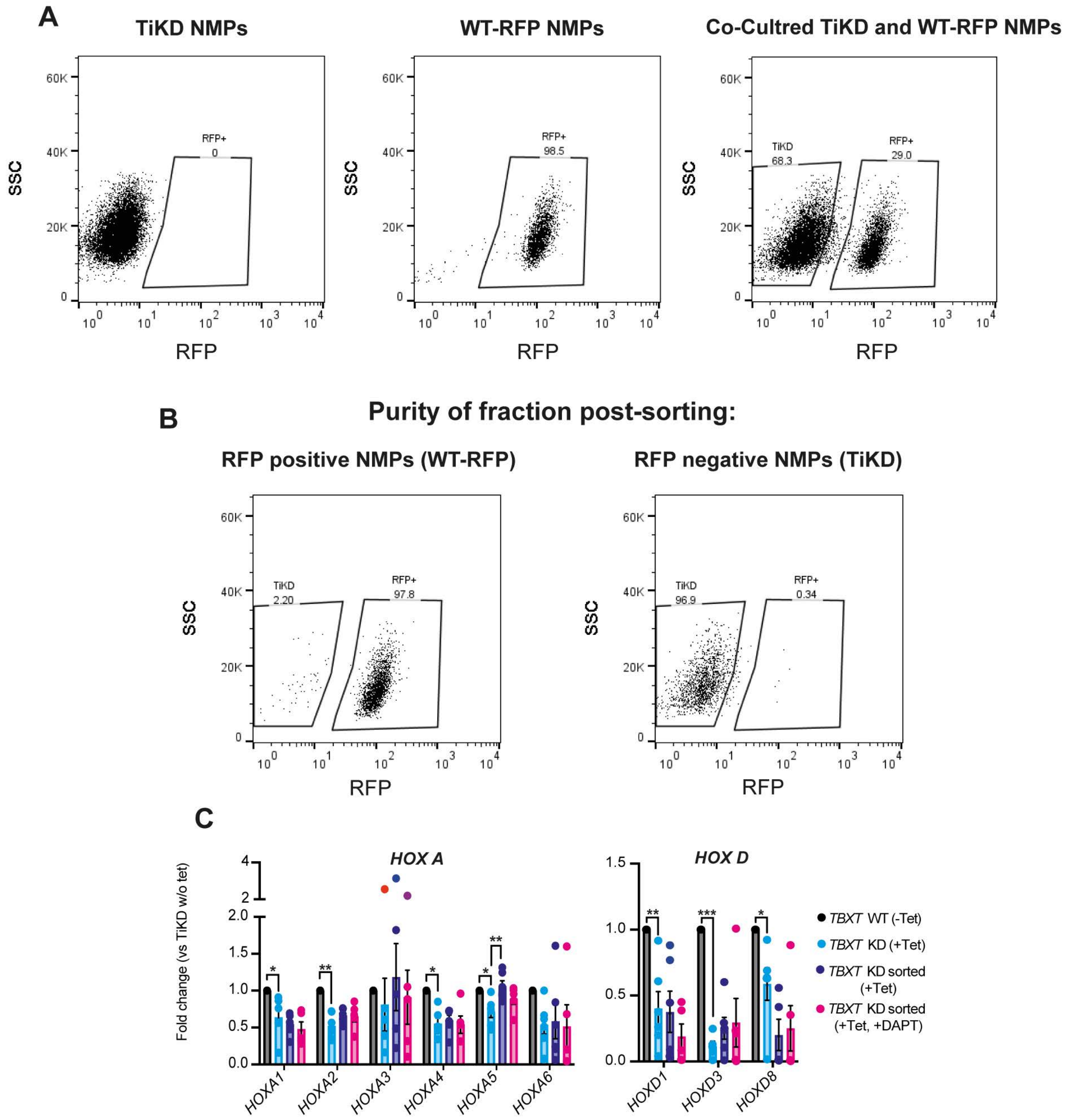

Fig. S3. Manipulation of TBXT expression and Notch signalling activity in co-cultures of hESC-derived NMPs. (A) FACS dot plots showing the fractions of RFP fluorescent reporter-positive cells in unlabelled TBXT knockdown (TiKD), wild type RFP (WT-RFP) hESC-derived NMPs and co-cultured (TiKD and WT-RFP) NMPs. (B) FACS dot plots showing the purity assessment following FACS of co-cultured NMPS into RFP negative (TiKD) and RFP positive (WT-RFP) fractions. (C) qPCR expression analysis of *HOX* genes belonging to paralogous groups A and D under the different experimental conditions depicted in Fig 2A. Error bars represent mean±s.e.m (n=3-6 independent experiments) *P≤0.05, **P≤0.01 ***P=≤0.001 (one sample t and Wilcoxon test (TiKD w/o Tet vs TiKD (+Tet)) or an unpaired two-tailed t.test (TiKD (+Tet) vs TiKD sorted (+tet) vs TiKD (+DAPT +Tet).

Development • Supplementary information

A

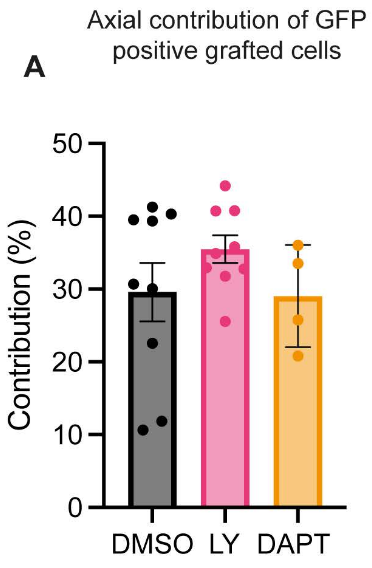

B

Uncorrected Fishers LSD T.Test: Intermediate phenotype

LY vs DMSO	Dorsal Neural Tube	Ventral Neural Tube	Floor Plate	Paraxial Mesoderm	Endoderm	Notochord
Rostral	ns	ns	ns	ns	*(0.0282)	ns
Middle	ns	ns	ns	ns	**(0.0054)	ns
Caudal	ns	ns	ns	ns	ns	**(0.0033)
Pre-progenitor	ns	ns	ns	ns	ns	**(0.0011)

Uncorrected Fishers LSD T.Test: Severe phenotype

LY vs DMSO	Dorsal Neural Tube	Ventral Neural Tube	Floor Plate	Paraxial Mesoderm	Endoderm	Notochord
Rostral	ns	***(0.0007)	ns	***(<0.0001)	ns	ns
Middle	ns	ns	***(0.0003)	**(0.0089)	ns	ns
Caudal	ns	ns	ns	ns	ns	**(0.0031)
Pre-progenitor	ns	ns	ns	ns	ns	***(<0.0011)

Uncorrected Fishers LSD T.Test: DAPT

LY vs DMSO	Dorsal Neural Tube	Ventral Neural Tube	Floor Plate	Paraxial Mesoderm	Endoderm	Notochord
Rostral	***(<0.0001)	***(0.0007)	ns	**(<0.0059)	ns	ns
Middle	ns	ns	**(0.0056)	ns	ns	ns
Caudal	ns	ns	ns	ns	ns	ns
Pre-progenitor	ns	ns	ns	ns	ns	ns

C

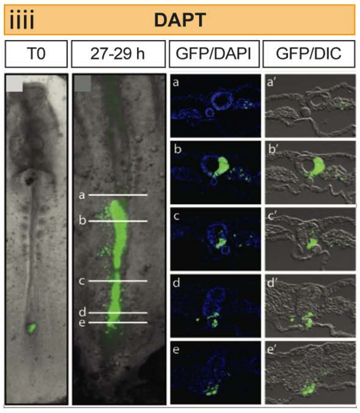

D

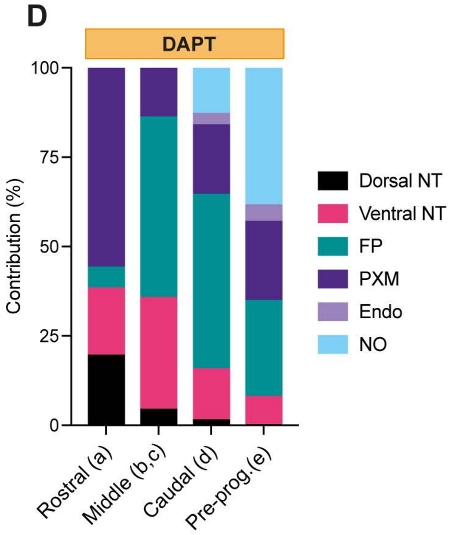

Fig. S4. Axial contribution of chick embryonic axial progenitors in the presence and absence of Notch signalling inhibition. (A) Percentage anterior-posterior embryonic axis colonised by cells from the NSB following DMSO, LY and DAPT treatment. Error bars indicate mean±s.e.m (DMSO n=9, LY n=9, DAPT n=4). ns (unpaired two-tailed t.test). (B) Table showing the statistical P-value results for the severe and moderate LY phenotype using a one-way ANOVA (Fisher's LSD test) (analysed sectioned embryos: DMSO n=9, LY severe n=4/9 and moderate n=5/9 and DAPT n=4). (C) Wholemount embryo at the time of receiving a NSB graft (T0) and the GFP contribution pattern following culture in the presence of the Notch inhibitor DAPT embryos after 27-29 hours following the graft. Transverse sections at the level of the white indicator lines (a, b, c, d, e) show the nuclear show the nuclear stain DAPI and GFP or DIC with GFP (a', b', c', d', e'). Images are representative of independent experiments (analysed sectioned embryos: DAPT = 4. (D) Quantification of the proportion (%) of GFP cells in transverse sections at position a (rostral), b and c (middle), d (caudal) and e (pre-progenitor, pre-prog.) contributing to axial and paraxial structures (dorsal neural tube (dorsal NT), ventral neural tube (ventral NT), floor plate (FP), paraxial mesoderm (somites rostrally and PSM caudally, PXM), endoderm (Endo) and the notochord (No) in DMSO and LY-treated.

Development • Supplementary information

Table S1. Antibodies used in this study

Available for download at
https://journals.biologists.com/dev/article-lookup/doi/10.1242/dev.202098#supplementary-data

Table S2. Primer sequences used in this study

Available for download at
https://journals.biologists.com/dev/article-lookup/doi/10.1242/dev.202098#supplementary-data

Development • Supplementary information
