# Supplementary material for: Notch signalling influences cell fate decisions and HOX gene induction in axial progenitors
Source: Development. 2024 Feb 12;151(3):dev202098. doi: 10.1242/dev.202098 (PMC10911136; doi:10.1242/dev.202098)
Supplement: Supplementary file 1 [file develop-151-202098_review_history.pdf]

# Notch signalling influences cell fate decisions and HOX gene induction in axial progenitors

Fay Cooper, Celine Souilhol, Scott Haston, Shona Gray, Katy Boswell, Antigoni Gogolou, Thomas J R Frith, Dylan Stavish, Bethany M James, Daniel Bose, Jacqueline Kim Dale and Anestis Tsakiridis
DOI: 10.1242/dev.202098

**Editor**: James Wells

**Review timeline**

| | |
|---|---|
| Original submission: | 16 June 2023 |
| Editorial decision: | 3 August 2023 |
| First revision received: | 1 December 2023 |
| Accepted: | 20 December 2023 |

---

**Original submission**

First decision letter

MS ID#: DEVELOP/2023/202098

MS TITLE: Notch signalling influences cell fate decisions and HOX gene induction in axial progenitors

AUTHORS: Fay Cooper, Celine Souilhol, Scott Haston, Shona Gray, Katy Boswell, Antigoni Gogolou, Tom Frith, Dylan Stavish, Bethany M James, Daniel Arun Bose, Jacqueline Kim Dale, and Anestis Tsakiridis

I have now received all the referees' reports on the above manuscript, and have reached a decision. The referees' comments are appended below, or you can access them online: please go to Bench Press and click on the 'Manuscripts with Decisions' queue in the Author Area.

As you will see, the referees express considerable interest in your work, but have some significant criticisms and recommend a substantial revision of your manuscript before we can consider publication. If you are able to revise the manuscript along the lines suggested, which may involve further experiments, I will be happy receive a revised version of the manuscript. Your revised paper will be re-reviewed by one or more of the original referees, and acceptance of your manuscript will depend on your addressing satisfactorily the reviewers' major concerns. Please also note that Development will normally permit only one round of major revision. If it would be helpful, you are welcome to contact us to discuss your revision in greater detail. Please send us a point-by-point response indicating your plans for addressing the referees' comments, and we will look over this and provide further guidance.

Please attend to all of the reviewers' comments and ensure that you clearly highlight all changes made in the revised manuscript. Please avoid using 'Tracked changes' in Word files as these are lost in PDF conversion. I should be grateful if you would also provide a point-by-point response detailing how you have dealt with the points raised by the reviewers in the 'Response to Reviewers' box. If you do not agree with any of their criticisms or suggestions please explain clearly why this is so.

Reviewer 1

© 2024. Published by The Company of Biologists under the terms of the Creative Commons Attribution License (https://creativecommons.org/licenses/by/4.0/).

*Advance summary and potential significance to field*

It has been reported that FGF and WNT signaling are required for NMP regulation. However, contribution of Notch signaling to NMPs remain unclear. The authors demonstrated that Notch signaling regulates cell fate decision and HOX gene expression in hESC-derived NMPs by pharmacological inhibition of NOTCH signaling and qRT-PCR. Furthermore, the authors indicated that inhibition of NOTCH signaling caused reduction of contribution to paraxial mesoderm derived from NMPs in chick embryos, revealing the function of Notch signaling in vivo.
I believe that the authors' work has high impact in the research field. However, there are still some points to be elucidated.

*Comments for the author*

Major points
The authors mainly analyzed gene expression by qRT-PCR but it cannot distinguish TBXT-single, SOX2-single and especially TBXT/SOX2-double positive cells. Important experiments (if not all) should be reanalyzed by immunofluorescence staining or fluorescent in situ hybridization.

1. The authors should indicate how NOTCHi affects NMP differentiation. Cell counts of TBXT single, SOX2 single and TBXT/SOX2 double-positive cells and double-negative cells in the condition of undifferentiated hESCs, DMSO-NMPs and NOTCHi-NMPs, is required.

2. The authors should indicate whether Notch signal components (such as receptors, ligands and RBPjk) is expressed in the undifferentiated hESCs, DMSO-NMPs and NOTCHi-NMPs. The authors described expression of Notch signal components in NMPs by citing transcriptome analysis in their previous works but only transcriptome analysis is not enough as reliable evidence to show expression of NOTCH signal components in NMPs. qRT-PCR in Fig S1 is supportive, but not suitable for analyses in heterogeneous population. Immunofluorescence staining or fluorescent in situ hybridization is required to distinguish TBXT single, SOX2 single and TBXT/SOX2 double-positive cells and double-negative cells.

3. The authors should indicate effect of NOTCH activation in NMPs such as NICD overexpression. In this manuscript, only NOTCH inhibition was done, and NOTCH activation experiments are required.

4. The authors should indicate NOTCHi effect after completion of NMP induction. Because DAPT treatment was started at the beginning of NMP induction, there are two possibilities: 1. DAPT inhibits differentiation from hESCs to NMPs. Undifferentiated ESCs (at least in mouse) are TBXT-negative/SOX2-positive and they are like NOTCHi-NMPs. 2. DAPT inhibits paraxial cell fate decision in bipotent NMPs. NOTCHi experiments before/after NMP induction should be distinguished.

Minor points
1. In Fig. 1A, the authors should explain why ROCK inhibitor is required.

2. In Fig. 1C, high magnification and merged picture are required to show whether HOXC9/TBXT/SOX2 are coexpressed in the same cells or not.

3. Line134
"… and data not shown"
Data should be indicated.

4. Line 143
"…a decrease in HOXC9, TBXT and SOX2 protein level…"
SOX2 protein level would be upregulated.

5. Line 179-181
Plausible explanation is required for Hes5 upregulation in TBXT KD with H9-RFP co-culture. Hes5 expression in TBXT KD with H9-RFP co-culture, is obviously higher than in the control (TBXT WT without Tet) that NOTCH signaling is ideally active as in the co-culture condition.

6.

© 2024. Published by The Company of Biologists under the terms of the Creative Commons Attribution License (https://creativecommons.org/licenses/by/4.0/).

Line 243: LY treatment (n=13)
Line 259: LY-treated embryos (“severe”; n=4/9)
Line 262: LY-associated “moderate” (n=5/9)
Sample number should be corrected.
For implant experiments, more sample number is required.

7. Line 259-262
The authors only described about caudal/pre-progenitor domains. The authors should describe about rostral and middle domains too.

8. Line 272-276
“Similar functional interactions between the two pathways have also been reported during the transition of axial progenitor-derived pre-neural and presomitic mesoderm cells toward spinal cord neurectoderm and somitic mesoderm respectively (Akai et al., 2005; Anderson et al., 2020; Diaz-Cuadros et al., 2020).”
The authors should explain how the results is similar to and, especially, different from the previous reports.
Akai et al., 2005 indicated that FGF-dependent Cash4 induce Delta1 in spinal cord development.
Anderson et al., 2020 indicated that Fgf4 maintains Hes7 oscillation in the presomitic mesoderm.
Diaz-Cuadros et al., 2020 indicated that FGF signal inhibition impair Hes7 oscillation in hiPSCS-derived PSM cells. These reports indicated NOTCH and FGF interaction, but not in the same manners.

9. Fig. 1A.
The authors should indicate asterisks and p-values in TBX6 and CDX2.

10. Fig. 3F.
The authors should indicate asterisks and p-values in SOX2, SOX1 and PAX6.

11. Fig. 3F.
TBXT expression should be analyzed.

12. Fig. 3G. H.
The authors should explain why FGF inhibition resulted in HES5 upregulation. FGF is usually required for induction of HES family genes under control of NOTCH signaling.

13. Fig. 4B and C.
The authors should indicate which sections in Fig. 4B are correspond to each domain in Fig. 4C.

14. MATERIALS AND METHODS
Catalogue numbers of commercially available materials should be indicated as readers can identify them.

15. Line 375
TBTX should be TBXT.

16. Line 383
mTESR would be mTeSR.

Reviewer 2

*Advance summary and potential significance to field*

In this manuscript, Cooper et al study the role of Notch signalling in the formation of axial progenitors through the use of both human ESCs as an in vitro model system and chick grafting experiments. The data demonstrate that Notch inhibition in the NMP population affects the Hox code of these progenitors and that it may also affect the progression of NMPs towards paraxial mesoderm.

© 2024. Published by The Company of Biologists under the terms of the Creative Commons Attribution License (https://creativecommons.org/licenses/by/4.0/).

Additionally, they show through some elegant and well controlled experiments that the effect of Notch inhibition on Hox genes can be rescued via non-cell autonomous means. The authors also provide evidence that Notch signalling promotes FGF signalling, which in turn can feedback and negatively regulate Hes5. Finally, they show through technically challenging grafting experiments that Notch signalling inhibition reduces the frequency at which transplanted axial progenitors contribute to paraxial mesoderm in an in vivo setting. These findings represents an important contribution to the development and stem cell field.

*Comments for the author*

It was enjoyable to read this manuscript. It is well written and in general, the figures are presented extremely well.

I have some suggestions to help with data interpretation. I encourage revision.

Main comments:
Figure 1: It is unclear if the reduction of TBXT, TBX6 in the Notch inhibition condition results in complete block in differentiation towards paraxial mesoderm, or delay. The data presented are at the NMP state. What happens at later timepoints? Are the proportions of neural vs mesoderm progenitors produced by NMPs altered following notch inhibition, relative to untreated NMPs? I raise this point as there are several places in the text where the authors conclude that the effect of Notch inhibition skews the balance towards a “pro-mesodermal” identity, at the expense of neural. Yet, in mouse ESC derived NMPs, at least the removal of Tbx6 results in a prolonged NMP state (c.f. Gouti et al 2017).
Indeed, the grafting experiments show that Notch inhibition alters the frequency at which the transplanted cells incorporate into paraxial mesoderm. However there is also an increase in the contribution towards notochord progenitors.
Thus, the in vitro system at a later time point may directly demonstrate a change in the balance between paraxial mesoderm vs spinal cord, to be able to direct state that. Alternatively, please consider amending these statements in the text.
Figure 3. The data presented suggests that FGF signalling levels can rescue the expression of Hox genes. Does this rescue the ability to produce paraxial mesoderm? If the authors can demonstrate this, this would also help to better clarify the model presented in H. At the moment it is unclear whether the effects of Notch inhibition on Hox expression can be uncoupled from neural vs mesodermal lineage decisions. Please also note that the model diagram implies that neural progenitors do not express Hox genes.

Minor comments:

1. Figure 1. It is difficult to interpret the qPCR data without having the relative levels of expression presented. I appreciate the efforts in broadly surveying the Hox locus, however, as currently presented, it’s unclear if all of the changes detected are relevant to present. Eg is HoxA10 expressed at very low levels and thus the reduction is minor? Given that this is a single time point I suggest focusing on the main targets that show the most robust expression.
This may also explain why the changes in Hoxc9 appear to be quite subtle by IF compared with the qPCR data. Similarly, in Figure 2, if the main effect is on the HoxB cluster, the remaining Hox genes assayed could be moved to the supplement.
2. Figure 1B. Could the authors double check if the reduction in TBX6 is also significant?
3. Figure 3B. The data for HES5 and Notch1 might be a repeat of the data in Figure S1A.
4. Figure 3H. The legend for this panel is missing.
5. Figure 4A. Please amend/check the cartoon summarises the experiment. Eg, no LY/DMSO treatment to the host?
6. Referring to line 126, and Figure S1A. Please amend the text – Hes5 appears to be the main target affected.

---

**First revision**

© 2024. Published by The Company of Biologists under the terms of the Creative Commons Attribution License (https://creativecommons.org/licenses/by/4.0/). 

Author response to reviewers' comments

To James Wells

We are pleased to resubmit our manuscript entitled “Notch signalling influences cell fate decisions and HOX gene induction in axial progenitors” (DEVELOP/2023/202098). We would like to thank the reviewers for their valuable feedback, which contributed significantly to the improvement of the manuscript. The revised version includes new data and text changes (highlighted in yellow), which address the issues raised.

Below is our point-to-point response to the suggestions/comments of the reviewers (in red italics):

***Reviewer #1***
*“I believe that the authors’ work has high impact in the research field”.*
We would like to thank the reviewer for their positive comments.

*“The authors mainly analyzed gene expression by qRT-PCR but it cannot distinguish TBXT- single, SOX2- single and especially TBXT/SOX2-double positive cells. Important experiments (if not all) should be reanalyzed by immunofluorescence staining or fluorescent in situ hybridization.*
*1. The authors should indicate how NOTCHi affects NMP differentiation. Cell counts of TBXT single, SOX2 single and TBXT/SOX2 double-positive cells and double-negative cells in the condition of undifferentiated hESCs, DMSO-NMPs and NOTCHi-NMPs, is required”.*
We have now included quantification of TBXT-, SOX2- and HOXC9- protein expressing cells emerging under different culture regimens, following immunolabelling and image analysis to address this point (see new **Figures 3F** and **S2B** in revised manuscript).

*“2. The authors should indicate whether Notch signal components (such as receptors, ligands and RBPjk) is expressed in the undifferentiated hESCs, DMSO-NMPs and NOTCHi-NMPs. The authors described expression of Notch signal components in NMPs by citing transcriptome analysis in their previous works, but only transcriptome analysis is not enough as reliable evidence to show expression of NOTCH signal components in NMPs. qRT-PCR in Fig S1 is supportive, but not suitable for analyses in heterogeneous population. Immunofluorescence staining or fluorescent in situ hybridization is required to distinguish TBXT single, SOX2 single and TBXT/SOX2 double-positive cells and double-negative cells”.*
To provide a better insight into the expression of Notch signalling components in NMPs we have now added new data (**Fig. S1** in revised manuscript) showing:

1) Time-course expression of Notch signalling-associated transcripts in the NMP- containing posterior growth region encompassing the caudal lateral epiblast/primitive streak in chick embryos from gastrulation to early somitogenesis stages.
2) Expression of Notch signalling-associated transcripts in the early somite-stage mouse embryonic posterior growth region.
3) ForceAtlas2 layouts of single-cell (k-nearest neighbour) kNN graphs overlaid with log-normalized transcript counts for key components of the Notch pathway from a published time-course single cell RNA-sequencing dataset obtained from human induced pluripotent stem cells differentiating toward NMPs and early presomitic mesoderm cells (Diaz-Cuadros et al., 2020).

We attempted immunofluorescence analysis in our *in vitro*-derived NMP cultures using several commercially available antibodies. Unfortunately, none of these antibodies were found to be reliable as they either resulted in a negative signal compared to secondary controls or non-specific localisation in 100% of cells.

*“3. The authors should indicate effect of NOTCH activation in NMPs such as NICD overexpression. In this manuscript, only NOTCH inhibition was done, and NOTCH activation experiments are required.”*
To address this, we attempted to over-activate Notch using a commercially available small molecule (Yhhu-3792), which was previously reported to activate the Notch signalling pathway (Morales et al., 2022). We found that at high concentrations of Yhhu-3729, NMPs were not viable, while at lower levels, Notch pathway components were actually down-regulated at the RNA level. Thus, we have not included these experiments in the manuscript. Our previous efforts to

© 2024. Published by The Company of Biologists under the terms of the Creative Commons Attribution License (https://creativecommons.org/licenses/by/4.0/).

electroporate chick embryonic NMPs with a NICD -overexpressing plasmid have also been unsuccessful as cells did not survive after electroporation. Other labs have also attempted this approach in the mouse and similarly failed as the cells do not survive (Val Wilson personal communication). We believe that such overexpression assays are difficult to perform and interpret and they would require delicate modulation of Notch levels to achieve a physiologically relevant increase in signalling activity.

*"4. The authors should indicate NOTCHi effect after completion of NMP induction. Because DAPT treatment was started at the beginning of NMP induction, there are two possibilities: 1. DAPT inhibits differentiation from hESCs to NMPs. Undifferentiated ESCs (at least in mouse) are TBXT-negative/SOX2-positive and they are like NOTCHi-NMPs. 2. DAPT inhibits paraxial cell fate decision in bipotent NMPs. NOTCHi experiments before/after NMP induction should be distinguished."*

This is an important question. Our data indicate that Notch inhibition does not impair exit from pluripotency as protein levels of both NANOG and OCT4 are downregulated to the same extent following culture using our three-day NMP induction protocol regardless of Notch inhibition (See new **Figures S2C, D** in revised manuscript). We have also included new data testing whether NOTCHi-NMPs can commit to a presomitic /paraxial mesoderm identity *in vitro* (**Fig. 1D-F** in revised manuscript) using previously published mesoderm-inducing conditions (High levels of FGF/WNT agonists). These show that both early and continuous/late treatment of NMPs with DAPT abolishes their ability to induce the PSM marker TBX6 suggesting an early reliance of nascent NMPs on Notch signalling to acquire mesoderm competence.

*"Minor points*
*1. In Fig. 1A, the authors should explain why ROCK inhibitor is required."*
We have now included the following sentence in the methods section (lines 420-421): to address this point: "Rho-associated coil kinase (ROCK) inhibitor Y-27632 2HCl (10 µM, Adooq Biosciences) was added for the first day of NMP induction, <u>as previously described, to aid survival following plating as a single cell suspension</u> (Frith et al., 2018; Gouti et al., 2017)."

*"2. In Fig. 1C, high magnification and merged picture are required to show whether HOXC9/TBXT/SOX2 are coexpressed in the same cells or not."*
We have added merged images and an inset magnification showing co-expression of HOXC9/TBXT/SOX2 proteins in NMP cells (new **Fig. 1C**). This is also complemented by the quantification of the same protein expression requested by reviewer 1 shown in **Fig. S2B** in the revised manuscript.

*"3. Line134 "... and data not shown". Data should be indicated".*
We have now included representative images of PAX6 and SOX1 protein expression in new **Figure S2E.**

*"4. Line 143 "...a decrease in HOXC9, TBXT and SOX2 protein level..." SOX2 protein level would be upregulated."*
We would like to thank the reviewer for spotting this mistake, which has now been corrected in the revised submitted version.

*"5. Line 179-181 Plausible explanation is required for Hes5 upregulation in TBXT KD with H9- RFP co-culture. Hes5 expression in TBXT KD with H9-RFP co-culture, is obviously higher than in the control (TBXT WT without Tet) that NOTCH signalling is ideally active as in the co- culture condition".*
We have previously shown that *in vitro*-derived TBXT knockdown NMPs exhibit significantly reduced levels of the non-canonical Notch ligand *DLL3* (see Supplementary file 1 in Gogolou et al. (2022)), which has been reported to act as a Notch signalling antagonist (Ladi et al., 2005). We speculate that this may be the reason for the observed Notch overactivation (reflected by the large increase in *HES5* levels) following co-culture of wild type and TBXT knockdown cultures. We have added a sentence in lines 198-199 in the main text commenting on this.

*"6. Line 243: LY treatment (n=13).*
*Line 259: LY-treated embryos ("severe"; n=4/9).*
*Line 262: LY-associated "moderate" (n=5/9).*

© 2024. Published by The Company of Biologists under the terms of the Creative Commons Attribution License (https://creativecommons.org/licenses/by/4.0/).

*Sample number should be corrected. ”*
We thank the reviewer for spotting this error. We have now corrected n numbers to show 9 embryos analysed.

*“For implant experiments, more sample number is required”.*
Unfortunately, at this time we are not able to repeat these technically challenging grafting experiments to increase our sample number. However, we have included new data from grafting experiments employing a second Notch Inhibitor DAPT (n = 4), which show the same result as the one observed with LY treatments (see **Fig. S4** in revised manuscript). We have also included presomitic mesoderm induction experiments using hESC-derived NMPs, which show the same phenotype (impaired presomitic/paraxial mesoderm commitment) in NOTCHi conditions (see above). Together, these data robustly demonstrate the impact of Notch inhibition on the mesoderm potential of NMPs.

*“7. Line 259-262*
*The authors only described about caudal/pre-progenitor domains. The authors should describe about rostral and middle domains too.”*
We have now updated the text to clearly describe our findings corresponding to all regions along the A-P axis (lines 279-283 in revised manuscript).

*“8. Line 272-276*
*“Similar functional interactions between the two pathways have also been reported during the transition of axial progenitor-derived pre-neural and presomitic mesoderm cells toward spinal cord neurectoderm and somitic mesoderm respectively (Akai et al., 2005; Anderson et al., 2020; Diaz-Cuadros et al., 2020).” The authors should explain how the results is similar to and, especially, different from the previous reports. Akai et al., 2005 indicated that FGF-dependent Cash4 induce Delta1 in spinal cord development. Anderson et al., 2020 indicated that Fgf4 maintains Hes7 oscillation in the presomitic mesoderm. Diaz-Cuadros et al., 2020 indicated that FGF signal inhibition impair Hes7 oscillation in hiPSCS-derived PSM cells. These reports indicated NOTCH and FGF interaction, but not in the same manners.”*
We have amended the text to reflect more accurately the findings of these papers and stressing that the relationship between NOTCH-FGF is cell type-/developmental context- specific (lines 299-301 in revised manuscript).

*“9. Fig. 1A.*
*The authors should indicate asterisks and p-values in TBX6 and CDX2.”*
We apologise for the confusion here. There was no statistically significant difference in the expression levels of TBX6/CDX2 in Fig. 1A and these lines were left in error. We have chosen to remove comparisons when they are not statistically significant.

*“10. Fig. 3F.*
*The authors should indicate asterisks and p-values in SOX2, SOX1 and PAX6.”*
Figure 3F (now **Fig. 3H**) has now been updated to include statistical analysis.

*“11. Fig. 3F.*
*TBXT expression should be analyzed.”*
We have not included TBXT expression analysis under FGF inhibition conditions here as this has been covered extensively in our recent manuscript (See Figure 5 in Gogolou et al. (2022)). However, we have added a sentence in the text (lines 231-232) to highlight this clearly: “…TBX6 expression was significantly reduced while the transcript levels of the pro-neural marker SOX2 increased (Fig. 3H) <u>while we have previously shown a reduction in TBXT expression under these conditions (Gogolou et al., 2022) “.</u>

*“12. Fig. 3G. H.*
*The authors should explain why FGF inhibition resulted in HES5 upregulation. FGF is usually required for induction of HES family genes under control of NOTCH signaling.”*
Previous work has shown that ERK1/2 acts as a negative regulator of γ-secretase, potentially mediating a feedback loop between Notch and FGF signalling (Jaroonwitchawan et al., 2016; Kim et al., 2006). We have included this sentence in the main text as a potential explanation of our finding (lines 243-245 in revised manuscript).

© 2024. Published by The Company of Biologists under the terms of the Creative Commons Attribution License (https://creativecommons.org/licenses/by/4.0/).

*“13. Fig. 4B and C.*
*The authors should indicate which sections in Fig. 4B are correspond to each domain in Fig. 4C.”*
We thank the reviewer for this suggestion. We have labelled the graph more clearly and have also included this information in the figure legend.

*“14.MATERIALS AND METHODS*
*Catalogue numbers of commercially available materials should be indicated as readers can identify them.”*
We have included all catalogue numbers in the Methods section where applicable.

*“15. Line 375*
*TBTX should be TBXT.”*
This has been corrected (line 394).

*“16. Line 383*
*mTESR would be mTeSR.”*
This has been corrected (line 390).

***Reviewer #2***
*“These findings represent an important contribution to the development and stem cell field. It was enjoyable to read this manuscript. It is well written and in general, the figures are presented extremely well. I have some suggestions to help with data interpretation. I encourage revision”.*
We would like to thank the reviewer for their support and valuable feedback.

*“Figure 1: It is unclear if the reduction of TBXT, TBX6 in the Notch inhibition condition results in complete block in differentiation towards paraxial mesoderm, or delay. The data presented are at the NMP state. What happens at later timepoints? Are the proportions of neural vs mesoderm progenitors produced by NMPs altered following notch inhibition, relative to untreated NMPs? … Thus, the in vitro system at a later time point may directly demonstrate a change in the balance between paraxial mesoderm vs spinal cord, to be able to direct state that”.*
This is an important point and a similar question was also raised by reviewer 1. To address this we have now added new data showing that:
1) Continuous culture of day 3 hESC-derived NMPs for a further 3 days in NMP-inducing conditions (i.e. FGF2 and CHIR) and in the presence of DAPT does not result in upregulation of TBXT/TBX6 suggesting that impaired induction of these markers is not simply due to delayed NMP specification/pluripotency exit/presomitic mesoderm differentiation (new **Fig. S2F-I**).
2) Directed differentiation of day 3 hESC-derived NMPs toward presomitic mesoderm using a previously published protocol (high levels of FGF/WNT signalling agonists) combined with either early or continuous NOTCH inhibition results in failure to induced TBX6 and an increase in SOX2 levels suggesting that early NOTCH activity is critical for imposing a pro-mesodermal character/mesodermal competence in nascent NMPs at the expense of a neural progenitor identity (new **Fig. 1E-F**). See also our response to reviewer 1 above.

*“Figure 3. The data presented suggests that FGF signalling levels can rescue the expression of Hox genes. Does this rescue the ability to produce paraxial mesoderm? If the authors can demonstrate this, this would also help to better clarify the model presented in H. At the moment it is unclear whether the effects of Notch inhibition on Hox expression can be uncoupled from neural vs mesodermal lineage decisions. Please also note that the model diagram implies that neural progenitors do not express Hox genes.”*
To address this, we have assayed the levels of TBX6/TBXT transcripts and found that an increase of FGF restores partly TBXT but not TBX6 expression (new **Fig. 3E-G**). These findings suggest that the FGF-NOTCH axis controls HOX gene expression in uncommitted TBXT+SOX2+ NMPs (see increase in the number of TBXT+SOX2+HOXC9+ CELLS in NOTCHi NMPs treated with higher levels of FGF2, new **Fig. 3F**) and irrespective of definitive presomitic mesoderm specification. We have updated our model in new **Fig. 3J** accordingly.

*“Minor comments:*
*“ I suggest focusing on the main targets that show the most robust expression. This may also*

© 2024. Published by The Company of Biologists under the terms of the Creative Commons Attribution License (https://creativecommons.org/licenses/by/4.0/).

*explain why the changes in Hoxc9 appear to be quite subtle by IF, compared with the qPCR data. Similarly, in Figure 2, if the main effect is on the HoxB cluster, the remaining Hox genes assayed could be moved to the supplement"*
To address this point, we have only included HOX genes that are robustly induced (> 10-fold) in day 3 NMPs compared to undifferentiated hESCs (new **Fig. 1G**). Moreover, we have moved the analysis of HOXA and HOXD gene expression from Fig. 2 to Fig S3, as suggested by the reviewer.

*"Figure 1B. Could the authors double check if the reduction in TBX6 is also significant?"*
Due to the large variation in *TBX6* levels observed in DMSO-NMP experiments, the reduction in *TBX6* expression following DAPT treatment is not statistically significant.
*"Figure 3B. The data for HES5 and Notch1 might be a repeat of the data in Figure S1A"*
The HES5/NOTCH1 expression data in the two figures have been obtained from independent experiments but to avoid confusion/repetition we have decided to remove them from the new **Fig. 3** and only include evidence of effective NOTCH inhibition in the new **Fig. S2A.**

*"Figure 3H. The legend for this panel is missing."*
This has been corrected.

*" Figure 4A. Please amend/check the cartoon summarises the experiment. Eg, no LY/DMSO treatment to the host?"*
We have updated **Fig. 4A** to make it clear that both donor and host chick embryos have received treatment before and after grafting.

*"Referring to line 126, and Figure S1A. Please amend the text - Hes5 appears to be the main target affected".*
The graph has been updated to show results on a linear axis - this visualises the downregulation of *NOTCH1* and *HES1* in a more appropriate way. Although these transcripts are not as dramatically downregulated as HES5 upon DAPT treatment, the reduction in their expression is statistically significant corresponding to approximately a 50% drop in transcript level.

**References**


Diaz-Cuadros, M., Wagner, D. E., Budjan, C., Hubaud, A., Tarazona, O. A., Donelly, S., Michaut, A., Al Tanoury, Z., Yoshioka-Kobayashi, K., Niino, Y., Kageyama, R., Miyawaki, A., Touboul, J., & Pourquié, O. (2020). In vitro characterization of the human segmentation clock. *Nature*, *580*(7801), 113-118. https://doi.org/10.1038/s41586-019-1885-9

Gogolou, A., Souilhol, C., Granata, I., Wymeersch, F. J., Manipur, I., Wind, M., Frith, T. J. R., Guarini, M., Bertero, A., Bock, C., Halbritter, F., Takasato, M., Guarracino, M. R., & Tsakiridis, A. (2022). Early anteroposterior regionalisation of human neural crest is shaped by a pro-mesodermal factor. *Elife*, *11*. https://doi.org/10.7554/eLife.74263

Jaroonwitchawan, T., Muangchan, P., & Noisa, P. (2016). Inhibition of FGF signaling accelerates neural crest cell differentiation of human pluripotent stem cells. *Biochemical and Biophysical Research Communications*, *481*(1), 176-181. https://doi.org/https://doi.org/10.1016/j.bbrc.2016.10.147

Kim, S. K., Park, H. J., Hong, H. S., Baik, E. J., Jung, M. W., & Mook-Jung, I. (2006). ERK1/2 is an endogenous negative regulator of the gamma-secretase activity. *Faseb j*, *20*(1), 157-159. https://doi.org/10.1096/fj.05-4055fje

Ladi, E., Nichols, J. T., Ge, W., Miyamoto, A., Yao, C., Yang, L. T., Boulter, J., Sun, Y. E., Kintner, C., & Weinmaster, G. (2005). The divergent DSL ligand Dll3 does not activate Notch signaling but cell autonomously attenuates signaling induced by other DSL ligands. *J Cell Biol*, *170*(6), 983- 992. https://doi.org/10.1083/jcb.200503113

Morales, R. A., Rabahi, S., Diaz, O. E., Salloum, Y., Kern, B. C., Westling, M., Luo, X., Parigi, S. M., Monasterio, G., Das, S., Hernández, P. P., & Villablanca, E. J. (2022). Interleukin-10 regulates goblet cell numbers through Notch signaling in the developing zebrafish intestine. *Mucosal Immunol*, *15*(5), 940-951. https://doi.org/10.1038/s41385-022-00546-3


© 2024. Published by The Company of Biologists under the terms of the Creative Commons Attribution License (https://creativecommons.org/licenses/by/4.0/).

Second decision letter

MS ID#: DEVELOP/2023/202098

MS TITLE: Notch signalling influences cell fate decisions and *HOX* gene induction in axial progenitors

AUTHORS: Fay Cooper, Celine Souilhol, Scott Haston, Shona Gray, Katy Boswell, Antigoni Gogolou, Thomas J R Frith, Dylan Stavish, Bethany M James, Daniel Bose, Jacqueline Kim Dale, and Anestis Tsakiridis

ARTICLE TYPE: Research Report

I am happy to tell you that your manuscript has been accepted for publication in Development, pending our standard ethics checks.

Reviewer 1

*Advance summary and potential significance to field*

I appreciate the authors' effort to improve the manuscript. The authors well answered all the points.

*Comments for the author*

Although overexpression experiments are important to underpin NOTCH signaling regulation, authors' findings are still significant to the research field. Finally, I recommend to publish the manuscript.

Reviewer 2

*Advance summary and potential significance to field*

In this manuscript, Cooper et al study the role of Notch signalling in the formation of axial progenitors through the use of both human ESCs as an in vitro model system and chick grafting experiments. The data demonstrate that Notch inhibition in the NMP population affects the Hox code of these progenitors and that it also affects the progression of NMPs towards paraxial mesoderm.
Additionally, they show through some elegant and well controlled experiments that the effect of Notch inhibition on Hox genes can be rescued via non-cell autonomous means. The authors also provide evidence that Notch signalling promotes FGF signalling, which in turn can feedback and negatively regulate Hes5. Finally, they show through technically challenging grafting experiments that Notch signalling inhibition reduces the frequency at which transplanted axial progenitors contribute to paraxial mesoderm in an in vivo setting. These findings represents an important contribution to the development and stem cell field.

*Comments for the author*

I thank the authors for their additions to the manuscript. I am satisfied with the responses and support publication.

© 2024. Published by The Company of Biologists under the terms of the Creative Commons Attribution License (https://creativecommons.org/licenses/by/4.0/).
